# Tunable potential well for plasmonic trapping of metallic particles by bowtie nano-apertures

**DOI:** 10.1038/srep32675

**Published:** 2016-09-26

**Authors:** Yu Lu, Guangqing Du, Feng Chen, Qing Yang, Hao Bian, Jiale Yong, Xun Hou

**Affiliations:** 1 Department of Electronic Science and Technology, State Key Laboratory for Manufacturing System Engineering and Key Laboratory of Photonics Technology for Information of Shaanxi Province, School of Electronics & Information Engineering, Xi’an Jiaotong University, Xi’an, 710049, PR China; 2 School of Mechanical Engineering, Xi’an Jiaotong University, Xi’an, 710049, PR China

## Abstract

In this paper, the tunable optical trapping dependence on wavelength of incident beam is theoretically investigated based on numerical simulations. The Monte Carlo method is taken into account for exploring the trapping characteristics such as average deviation and number distribution histogram of nanoparticles. It is revealed that both the width and the depth of potential well for trapping particles can be flexibly adjusted by tuning the wavelength of the incident beam. In addition, incident wavelengths for the deepest potential well and for the strongest stiffness at bottom are separated. These phenomena are explained as the strong plasmon coupling between tweezers and metallic nanoparticles. In addition, required trapping fluence and particles’ distributions show distinctive properties through carefully modifying the incident wavelengths from 1280 nm to 1300 nm. Trapping with lowest laser fluence can be realized with 1280 nm laser and trapping with highest precision can be realized with 1300 nm laser. This work will provide theoretical support for advancing the manipulation of metallic particles and related applications such as single-molecule fluorescence and surface enhanced Raman spectroscopy.

In recent years, stable plasmonic trapping of the metallic particles has stimulated huge interest due to the available strongly enhanced nanoscale e-fields^1^,^2^,^3^, which has the potential applications such as single-molecule fluorescence and surface enhanced Raman spectroscopy^4^,^5^. Compared with other methods such as self-assembly and lithography^6^,^7^ that bears high complexity with multi-steps, plasmonic trapping of metallic nanoparticles can be more effective and flexible in acquiring narrow nanoscale e-fields between metallic nanoparticles and trapping instruments, namely, plasmonic tweezers^2^,^8^,^9^. The trapping potential wells of plasmonic tweezers have been proved to depend on several factors such as the geometry structures of tweezers as well as the wavelength of incident beams^10^,^11^,^12^. The majority of previous works have focused on the design of tweezers geometry structures such as nano-apertures, dipole nano-antennas and nano-plates for obtaining desired potential wells^1^,^3^,^10^,^11^. Theoretical investigation on tuning plasmonic trapping through incident beam wavelength, however, is still a less developed topic^12^,^13^. Investigations on wavelength trapping without modifying tweezers structures hold potentials in providing more flexible methods which could lead to less thermal effect to the surroundings, especially to the biological specimens, in potential applications^14^,^15^. In addition, compared with that for trapping dielectric particles, the trapping forces in the plasmonic trapping of metallic particles are even more sensitive to the incident wavelength due to the strong coupling mechanism between metallic nanoparticles and plasmonic optical tweezers^16^,^17^,^18^. Correspondingly, the tunabilities of plasmonic trapping of metallic nanoparticles with respect to the varying incident wavelength, for example, the modifications of required trapping fluence and average displacement of trapped particles, especially deserve to be investigated either theoretically or experimentally. As a result, it becomes necessary to have a systematic investigation on the tunabilities of plasmonic trappings via modifying the incident wavelength, which could provide sufficient support for the designing of the plasmonic trapping systems, choosing the optimized incident wavelength and advancing the related applications.

In this paper, the plasmonic trapping forces and the shape of the trapping potential wells depending on the wavelength of incident beams are investigated based on the finite element method (FEM). Bowtie nanoaperture, an effective structure for plasmonic trapping proved by previous works^19^,^20^, is applied to trap metallic nanoparticles within nanoscales. Prominent tunabilities of potential well, such as depth, width and stiffness at potential well bottom, were checked. Interestingly, incident wavelength for the deepest potential well and for the strongest stiffness at bottom show prominent difference. These phenomena observed in the simulation originate from the strong plasmon coupling between gold nanoparticles and plasmonic optical tweezers. In addition, the modification of trapping fluence and distribution of the trapped particles tuned by incident wavelength is investigated based on the Monte Carlo simulation.

## Results

**Figure 1:**
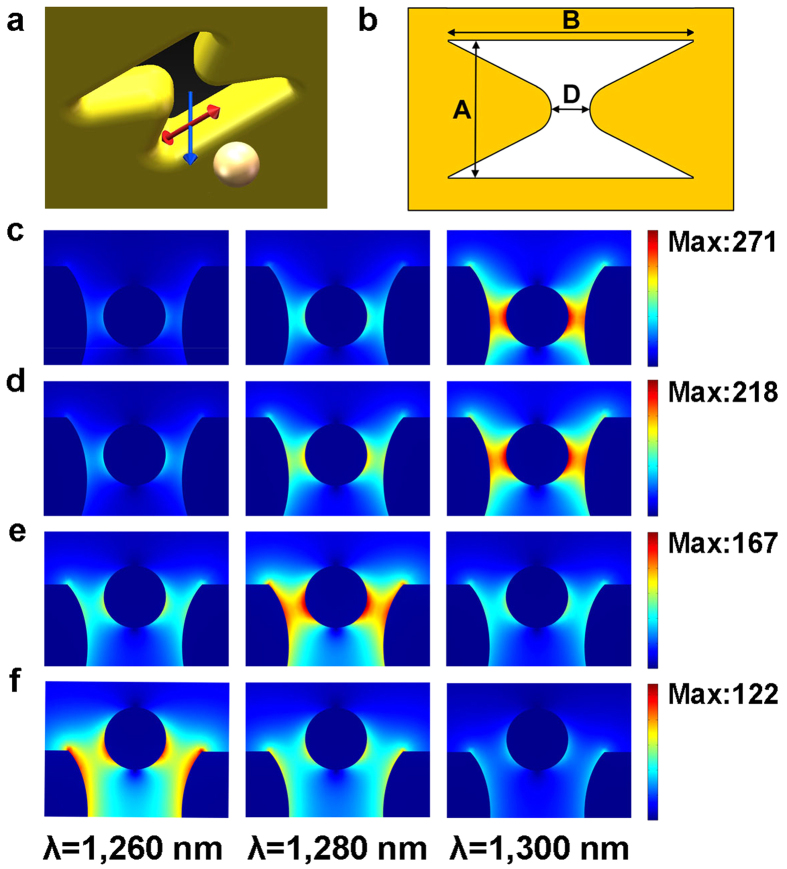
Schematic model for FEM simulation and e-field enhancement distribution. (**a**) The scheme of the plasmonic tweezers and the nanoparticle. The blue arrow denotes the incident direction and the red one demotes the polarization direction. The direction of the incident laser is vertical to the surface of the gold film where the bowtie nano-aperture (BNA) is milled. The incident light is polarized along the direction where the bowtie dimmer arranges. The diameter of the gold nanparticle is 50 nm. (**b**) The geometry of the BNA, the thickness of the gold film is 100 nm, A = 180 nm, B = 280 nm, D = 70 nm. (**c**–**f**) The e-field distribution under different incident wavelength with the distance between the nanoparticle and the tweezers of (**c**) 10 nm, (**d**) 20 nm, (**e**) 40 nm, (**f**) 60 nm.

The scheme of the bowtie nano-aperture has been shown in Fig. 1(b), in which the detailed geometry parameter has been given. It has been proved by the previous work that the great localized and enhanced e-field exists in the center of bowtie dimmers, enabling to generate enough e-field gradients to trap nanoparticles within nanoscales. The plasmon resonance modes in metallic nano-apertures, proved as Fabry-Pérot-like interfering of TE_10_ mode propagation wave, strongly depend on the aperture geometry^20^. Through modifying apertures sizes or shapes, the wavelength for plasmonic trapping can be flexibly tuned. The e-field enhancement distribution with respect to the spatial arrangement of the particle-tweezers system and the incident wavelength is shown in Fig. 1(c–f). When the displacement of the nanoparticle to the center of the tweezers is 10 nm and 20 nm, shown in Fig. 1(c,d), the maximum e-field enhancement is acquired at the wavelength of 1300 nm. When the displacement increases from 40 nm to 60 nm, the wavelength with respect to the maximum e-field enhancement exhibits a shift from 1280 nm to 1260 nm. The displacement dependence of resonance wavelength could be attributed to the coupling between the metallic nanoparticle and the optical tweezers. The strongly enhanced plasmon coupling, which exists when the nanoparticle is in vicinity of the center of tweezers, leads to the increase of the resonance wavelength of the entire particle-tweezers system and highly enhanced e-field under longer incident wavelength. Correspondingly, as the nanoparticle get away from the tweezers, the strength of the coupling decreases, leading to prominent blue shift of the resonance wavelength of the particle-tweezers system. As a result, highly enhanced e-field could be acquired with shorter wavelength.

**Figure 2:**
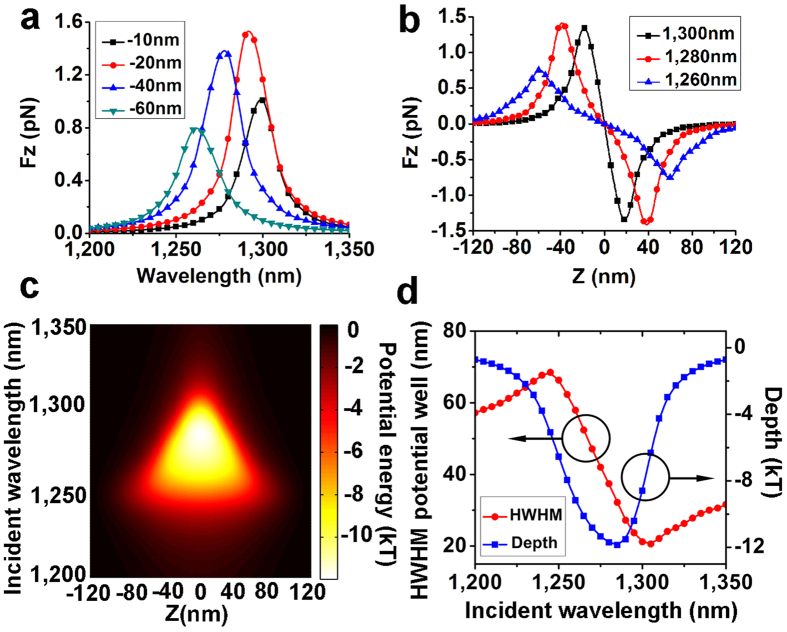
Trapping forces and trapping potential well. (**a**) The trapping force spectrum as a function of laser wavelength with different locations of the gold nanoparticles along Z axis (Z coordinates). (**b**) The trapping forces depending on the location of the nanoparticle under different incident wavelength. (**c**) Trapping potential energy depending on the varying incident wavelength. The Z direction is vertical to the surface of the gold film and the original point is in the center of the tweezers. (**d**) Half width at half maximum (HWHM, ◾) and depth (⦁) of potential well depending on incident wavelength. The laser fluence applied in this figure is 4 × 10^8^ W/m^2^.

The trapping forces spectrum as a function of laser wavelength for different locations of gold nanoparticles is shown in Fig. 2(a). The wavelength with respect to the strongest trapping force shows an evident blue-shift with increasing distance between nanoparticles and the center of tweezers. As the distance increases from 10 nm to 60 nm, the wavelength with respect to the strongest trapping force decreases from 1300 nm to 1260 nm. This observed blue-shift could be attributed to the weakening of the coupling between the plasmon of the particles and the tweezers. The wavelength dependence of the relationship between the location of the nanoparticle and the trapping force is also revealed as shown in Fig. 2(b). The displacement of particle to the center of tweezers with respect to the strongest trapping force exhibits an evident increase with the decreasing of incident wavelength from 1300 nm to 1260 nm. The strongest trapping force exists with a displacement of nearly 20 nm at the laser wavelength of 1300 nm. As the incident wavelength decreases to 1260 nm, however, the displacement with respect to the strongest trapping force extends to nearly 60 nm. When the particle is in vicinity of tweezers, the resonance wavelength of the particle-tweezers system approximates to 1300 nm. As a result, under the incident wavelength of 1300 nm, the strongest trapping force exists when the distance between the particle and the tweezers is 20 nm. When the particle escapes away from tweezers, the particle-tweezers system is off the resonance state under wavelength of 1300 nm due to the decrease of the coupling strength and the blue shift of the resonance wavelength. As a result, the trapping force drops quickly. Similarly, when the distance between the particle and the tweezers is small, the particle-tweezers system is in off-resonance state under the wavelength of 1260 nm because the resonance wavelength of the entire system is a much longer one. As the nanoparticle gradually escapes from the tweezers, the particle-tweezers system is approaching to the resonance state. As a result, the strongest force exists at the distance of 60 nm under the wavelength of 1260 nm. More detailed information about particle displacement for maximum trapping forces depending on incident wavelengths has been shown in supplementary figure, Fig. S1 on line. It can be seen from Fig. S1 that when decreasing incident wavelengths from 1330 nm to 1250 nm, the displacement for maximum trapping forces show so evident increase from 10 nm to 70 nm. The modification of the trapping force under different incident wavelengths would definitely lead to the modification of the shape of the potential energy well, which is shown in Fig. 2(c). With increasing the incident wavelength, the area corresponds to considerable depth of the potential well (bright area) shows an evident decreasing, indicating the rapidly decreasing of potential well width. More detailed information can be seen from the wavelength dependent half width at half maximum (HWHM) potential well shown in Fig. 2(d). The HWHM potential well shows the same trend as the displacement for maximum trapping force depending on incident wavelength, which is not hard to understand when considering the quasi-Gaussian shape of potential well. With the incident wavelength ranging from 1250 nm to 1300 nm, the HWHM decreases from 69 nm to 21 nm. For a longer wavelength, the trapping force, namely, the gradient of the trapping potential well, reaches maximum at a smaller distance between particles and tweezers and then drops quickly when the distance increases. As a result, the rapid dropping of the trapping force leads to the narrow trapping potential well. As the incident wavelength decreases, however, the distance between particles and tweezers with respect to the strongest trapping force increases. As a result, the width of the trapping potential shows an evident increase. Interestingly, the HWHM increases slightly when the incident wavelength is below 1250 nm and above 1300 nm, which could be attributed to the too short or too long incident wavelength which beyond the resonance wavelength of the particle-tweezers system all the time. The tunabilities of the depth of the potential well are also shown in Fig. 2(c,d). The deepest potential well near to minus 12 kT can be acquired with the incident wavelength of 1280 nm and the depth of potential can be controlled below minus 6 kT with the incident wavelength varying from 1240 nm to 1310 nm. Considering that stable trapping can be realized when the depth of potential well is below minus 10 kT^1^, it is possible to realize stable trapping with a laser fluence no higher than 8 × 10^8^ W/m^2^ (two times as that used in Fig. 2) by tuning the incident wavelength within the range from 1240 nm to 1310 nm. When the incident wavelength reaches to 1200 nm or 1350 nm, the potential well becomes ignorable. Supplementary figure, Fig. S2 on line shows the different groups of wavelength dependent potential well depth with difference bowtie nano-aperture sizes. Incident wavelength for deepest potential well evidently increases when enlarging the aperture size, which accords to the previous works^20^.

**Figure 3:**
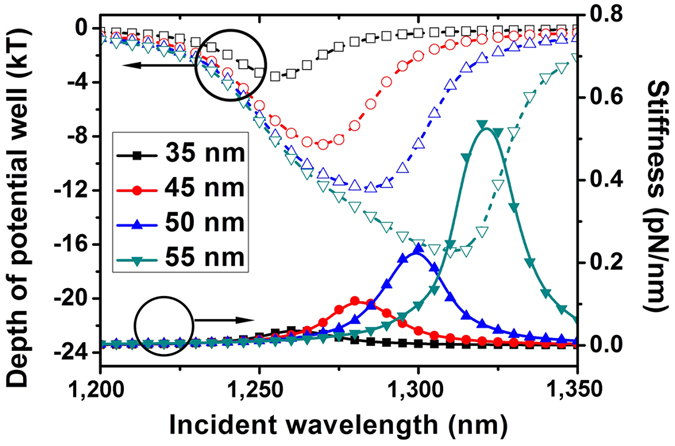
Wavelength dependent potential well depth (open dots) the stiffness at the center of tweezers (solid dots) with different metallic nanoparticle diameters of 35 nm (◾), 45 nm (⦁), 50 nm (▴) and 55 nm (▾). The laser fluence applied in this figure is 4 × 10^8^ W/m^2^. The wavelength dependence of potential well’s half width for maximum (HWHM) with different particle sizes is shown as figure R3 as supplementary information.

The Wavelength dependent potential wells depth (open dots) the stiffness at the center of tweezers with different metallic nanoparticle diameters of 35 nm, 45 nm, 50 nm and 55 nm have been shown in Fig. 3. With increasing metallic particle size, the incident wavelength for maximum potential well depth shows evident red shift from 1255 nm to 1310 nm. The corresponding deepest potential well also increases from minus 3.6 kT to minus 16.5 kT with increasing particle size. The increasing size of metallic particle could enhance its plasmon resonance and finally strengthen the plasmon coupling with plasmonic tweezers, leading to the red shift of the optimal incident wavelength for the deepest potential well. For the particles with diameters over 45 nm, potential deeper than minus 8 kT can be acquired with input laser intensity of 4 × 10^8^ W/m^2^. So stable trapping can be realized with input laser intensity of 5 × 10^8^ W/m^2^ with potential wells deeper than minus 10 kT. For the nanoparticles with diameters of 35 nm, however, the depth of potential well is minus 3.6 kT so laser fluence needs to be increased over 1 × 10^9^ W/m^2^ to realize stable trapping. In such condition, the better way to solve such problem may be modifying the geometry of our tweezers, for example, decreasing the gap (parameter D) to achieve stable trapping with lower incident fluence rather than increasing laser fluence to avoid damage of metallic nanostructures or surroundings. The maximum stiffness at potential well bottom increases rapidly with increasing particle size. The corresponding incident wavelength also shows evident red shift from 1260 nm to 1320 nm. With the particle size increasing, the plasmon coupling becomes stronger, leading to the increasing of plasmon resonance wavelength and resonance strength. Another interesting phenomenon is that the wavelength for the maximum stiffness and the maximum potential depth shown is evidently separated. For example, for the particle with diameter of 50 nm, the incident wavelength for maximum potential well depth is 1280 nm for the strongest stiffness is 1300 nm. Incident wavelength for maximal stiffness corresponds to the resonance wavelength of the particle-tweezers system when nanoparticle is in vicinity to the bottom of potential well. The strongest trapping forces also exist at that point. As the nanoparticle escape away from the center, trapping force drops rapidly due to the off-resonance states. As a result, the deepest potential well is not achieved in this case.

**Figure 4:**
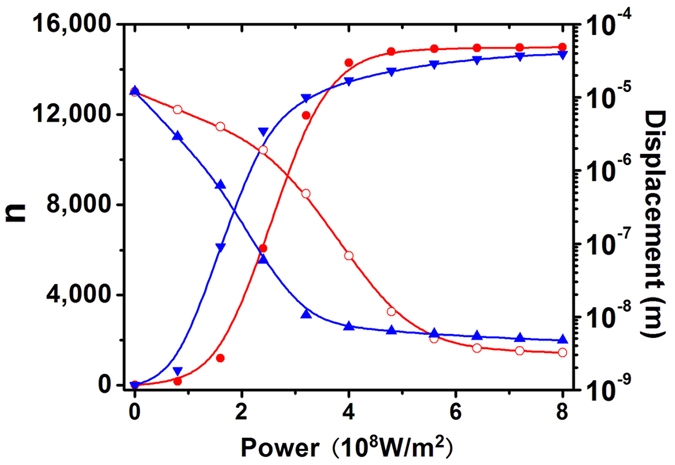
Average displacement of the simulated particles. (▴ for data acquired by 1280 nm laser and ○ for data acquired by 1300 nm laser) and the number of the nanoparticle trapped in with a displacement to the center of tweezers below 15 nm (▾ for data acquired by 1280 nm laser and ⦁ for data acquired by 1300 nm laser). The particle size is 50 nm. The media temperature applied in the simulation is 300 kT.

In Monte Carlo simulation, the average displacement and the number of the trapped particles depending on the fluence of the incident laser have been shown in Fig. 4. The wavelength of 1280 nm and 1300 nm, which corresponds to the incident wavelength for deepest potential well and for the strongest stiffness are applied, respectively. With increasing laser fluence, the average displacement of the nanoparticles irradiated by 1280 nm laser shows an evident decrease. When the laser flunece reaches to 3.2 × 10^8^ W/m^2^, the average displacement can be concentrated below 10 nm. With continuously increasing laser fluence, however, the average displacement does not exhibit an evident decrease and is still larger than 4.5 nm. At the incident laser of 1300 nm, the average displacement can be controlled below 10 nm only when the fluence is over 5 × 10^8^ W/m^2^. When the fluence reaches to 8 × 10^8^ W/m^2^ the average displacement can be controlled below 3 nm. When the laser fluence is lowered, it is the depth of the potential well that plays a key role in the trapping of nanoparticles. As a result, the stable trapping of nearly all the particles can be firstly realized with 1280 nm laser due to its deepest potential well. With increasing the laser fluence, both of the potential wells acquired by 1280 nm laser and 1300 nm laser are deep enough to assure a stable trapping. Correspondingly, it is the stiffness at the bottom of potential and width of the potential well that play key roles and nanoparticles are confined more efficiently by 1300 nm laser due to the stronger stiffness than 1280 nm laser. Similar phenomenon can be also observed when considering the total number of the particles trapped with the displacement below 15 nm. In low fluence, compared with the 1300 nm laser, more particles are trapped with a displacement below in vicinity to the center of tweezers by 1280 nm laser. When the fluence is over 4 × 10^8^ W/m^2^, however, the particle is more easily to be trapped in vicinity to the center of tweezers by the 1300 nm laser.

**Figure 5:**
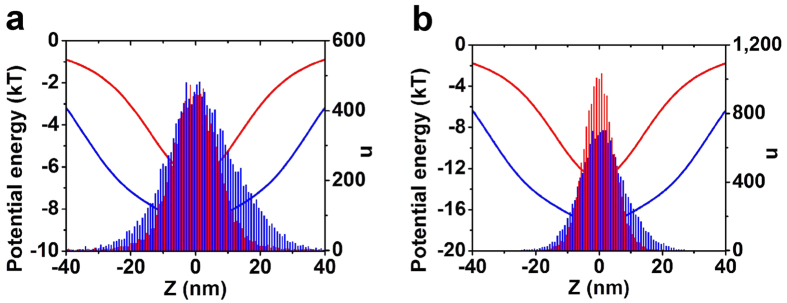
Potential well and number distribution histograms of the trapped particles with laser fluence of (**a**) 2.8 × 10^8^ W/m^2^ and (**b**) 5.6 × 10^8^ W/m^2^. The blue line and bars represent the simulated data acquired by 1280 nm laser and the red line and bars represent the simulated data acquired by 1300 nm laser. The particle size is 50 nm. The media temperature applied in the simulation is 300 kT.

The number distribution histograms of trapped particles and the shape of trapping potential wells under the laser power of 2.8 × 10^8^ W/m^2^ and 5.6 × 10^8^ W/m^2^ are shown in Fig. 5(a,b), in which the wavelength of 1280 nm and 1300 nm are applied. With the fluence of 2.8 × 10^8^ W/m^2^, the depth of the potential well is minus 8.7 kT for the 1280 nm laser and minus 6.5 kT for the 1300 nm laser. As a result, the total number of the trapped particles form −40 nm to 40 nm is 14778 for the 1280 nm laser and 10101 for the 1300 nm laser. Nearly all of the nanoparticles can be trapped with 1280 nm laser and only 67% of nanoparticles can be trapped by 1300 nm laser. The trapped particles are more concentrated to the center of tweezers when irradiated by the 1300 nm laser, which could also be attributed to the strong stiffness and narrow potential well width existing in long wavelength range. At the fluence of 5.6 × 10^8^ W/m^2^, the depth of the potential well is minus 17.5 kT for the 1280 nm laser and minus 13 kT for the 1300 nm laser, correspondently, 15000 particles are trapped for 1280 nm laser and 14998 particles are trapped for 1300 nm laser. Nearly all of the particles can be trapped with both kind of wavelength because both of the potential wells are deep enough to realize stable trapping. Compared with 1280 nm laser, much more particles could concentrate around the center of the tweezers, indicating a much more precise control of the trapped particles.

## Discussions

Conclusion can be got from Fig. 1(c–f) that the resonance states in the particle-tweezers system would be affected by the spatial arrangement of metallic nano-particle and tweezers due to the strong plasmon coupling mechanism. The displacements of the nanoparticles with respect to the resonance states of particle-tweezers system vary evidently from 10 nm to 60 nm when modifying the wavelength of the incident beam from 1260 nm to 1300 nm. Due to such strong and highly sensitive plasmon coupling mechanism, plasmonic trapping forces and potential well exhibit strong tunabilities when modifying the wavelength of the incident beam, which has been shown in Fig. 2. The geometry of the potential well, for example, the width and the depth, can be tuned with the varying wavelength of the incident beam. The width of the potential well decreases evidently with the incident wavelength increasing from 1250 nm to 1300 nm because longer wavelength corresponds to a resonance state, or for the same matter, the strongest trapping force, with a smaller displacement of nanoparticle. The depth of the potential well is considerable with the wavelength of incident beam varying from 1240 nm to1310 nm and the deepest potential well can be acquired with the incident wavelength of 1280 nm. The results indicate that stable trapping can be realized with low input power in a wide wavelength range. The strong coupling mechanism between nanoparticles and plasmonic tweezers can be also revealed when investigating wavelength dependence of potential well depth and stiffness with difference particle sizes, shown in Fig. 3. With the particle size increasing, incident wavelengths for both maximum potential well depth and for maximum stiffness at bottom show evident red shift. These results can help for choosing suitable wavelength for trapping particles with different sizes. In addition, the incident wavelengths for maximum potential well depth and for maximum stiffness at bottom show evident difference. Considering that potential well depth relates to the trapping time and trapping probabilities and stiffness at potential well bottom relates to the confine of particles at potential well bottom, the separation of incident wavelength for maximum potential well depth and for maximum stiffness at bottom will affect trapping properties.

It is revealed by Monte Carlo simulation that the tunabilities of the potential well width, depth and stiffness at bottom by switching the wavelength of incident beam will lead to the modifications of trapping properties to a large extent. Conclusion can be drown from Fig. 4 that two distinctive methods to advance the optic trapping of metallic particles, low-fluence induced trapping and high-precision trapping, can be realized by respective wavelengths. When 1280 nm laser is applied, the trapping under lowest fluence can be realized due the deepest potential well, on the other hand, at the cost of pulling down the confine of nanoparticles at the bottom. Precise controlling of the trapped particles can be realized by 1300 nm laser due to the great stiffness at potential well bottom, however, with high fluence to reach the necessary depth of the potential well. More detailed results have been shown in Fig. 5. The modification of trapped particles’ number distribution histograms is also evident when switching the incident wavelength. Although almost all the particles can be trapped by 1280 nm laser firstly when the laser intensity is low, more effective nanoparticle confine at potential well bottom can be acquired by 1300 nm laser. The trapped number is mainly determined by the depth of the potential well and the distribution of the trapped particles is mainly determined by the width of the potential well. The 1280 nm laser with the deepest potential well should be applied when only the trapping number needs considering. When the confines of trapped particles, namely, trapping precisions, should be considered, however, it is better to apply the 1300 nm laser to make trapped particles more approximate to the center of tweezers.

## Methods

The plasmonic optic tweezers (shown in Fig. 1(a)) is a bowtie nano-aperture (BNA) milled on a gold film with 100 nm thickness, the exact parameters of the geometry of which is shown in Fig. 2(b). The direction of the incident laser is vertical to the surface of the gold film where bowtie nano-aperture (BNA) is milled and the incident light is polarized along the direction where bowtie dimmer arranges. The diameter of the gold nanparticle is 50 nm. In this paper, the wavelength dependent trapping forces can be acquired through finite element method (FEM). E and H fields are calculated by FEM software itself in which a 3D simulation RF mode was built. A perfect matching layer (PML) is set outside of geometrical bowtie nano-aperture (BNA) and metallic nanopaticle. The scattered light from the bowtie is totally absorbed through the PML in the far field. The boundary condition at the interface between the BNA, nanoparticle and water medium is treated as continuous one. Then the BNA, nanoparticle and water medium and the PML zone are divided into many small tetrahedron meshes for the 3D areas and triangle meshes for boundaries, the smallest length of which could reach to 10 nm. A Helmholtz equation is built on the meshed geometry for describing the near-field scattering process. The Helmholtz equation is then discretized at every mesh points to form a large sparse matrix. Finally, we obtained the numerical solutions of the Helmholtz equation via solving the built matrix using FEM. The refractive index of the media is 1.33, corresponding to that of water, and the wavelength dependent dielectric constants of the gold can be acquired based on the classic Drude model^21^. The expression of trapping forces can be shown as^22^:




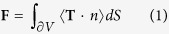




Here, 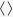 donates the time average, 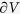
donates the surface of nanoparticle and **T** is the Maxwell’s stress tensor, which can be shown as:




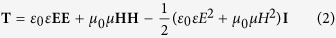




Here, **I** denotes the unit matrix. In Monte Carlo section, through which the deviation to the center of the tweezers and the number distribution of nanoparticles can be numerically acquired, an overdamping form of Brownian motion equation is adopted^23^. The exact expression, where an extra trapping force is induced, can be shown as:




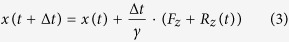




In eq. 3, *γ* = *6πrη* and *η* is the viscosity of water acquired in ref. 23. *F*_*z*_ is the trapping force on the z direction and *R*_*z*_*(t)* is the random forces with a standard deviation of 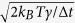. *k*_*B*_ is the Boltzmann constant and Δ*t* is selected to be *10 m/γ*. In our model, the Monte Carol simulation runs repeatedly for 15000 times. The original point of the nanoparticle is in the center of the tweezers and the total time of the single simulation is 10 s.

## Additional Information

**How to cite this article**: Lu, Y. *et al*. Tunable potential well for plasmonic trapping of metallic particles by bowtie nano-apertures. *Sci. Rep.* **6**, 32675; doi: 10.1038/srep32675 (2016).

## Supplementary Material

Supplementary Information
