# Supplementary material for: Tunable potential well for plasmonic trapping of metallic particles by bowtie nano-apertures
Source: Sci Rep. 2016 Sep 26;6:32675. doi: 10.1038/srep32675 (PMC5036050; doi:10.1038/srep32675)
Supplement: Supplementary Information [file srep32675-s1.pdf]

Tunable potential well for plasmonic trapping of metallic particles by bowtie nano-apertures

Yu Lu, Guangqing Du, Feng Chen*, Qing Yang*, Yan Ou, Jiale Yong and Xun Hou

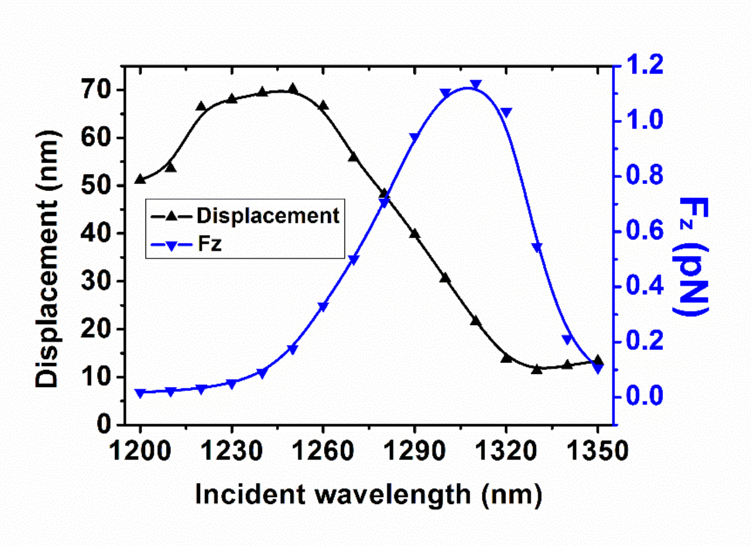

Figure. S1. The displacements for the maximum trapping force changing with incident wavelength (▲) and magnitude of the corresponding force (▼). The laser influence is 4×10^8 W/m^2 and the geometry parameters of the bowtie nanoaperture and particle are what applied in figure.2 in the manuscript.

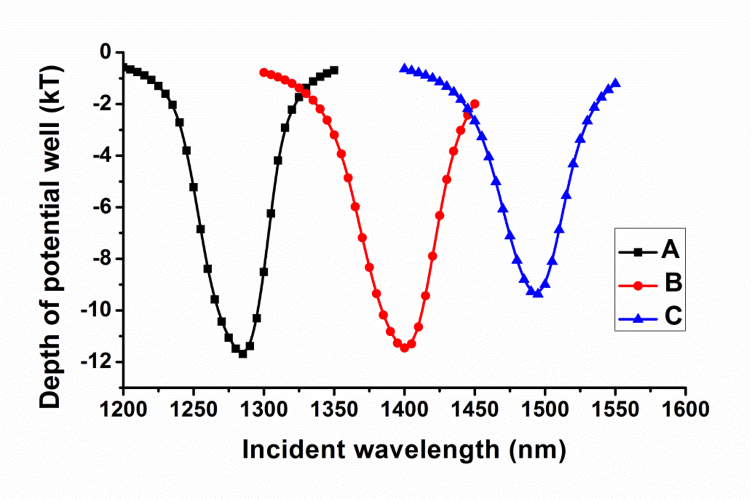

Figure S2. The wavelength dependent potential well depth with different bowtie nano-aperture sizes of A, B and C. A represents the parameters applied in **figure.1 (b)** in manuscript. B represents the size 1.15 times as that applied in **figure.1 (b)** and C represents the size 1.3 times as that applied in **figure.1 (b).** The laser influence is 4×10^8 W/m^2 and the diameter of nanoparticle is 50 nm.

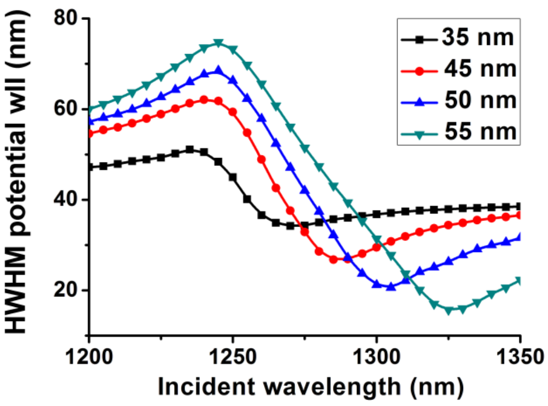

Figure S3. Wavelength dependent half width for half maximum (HWHM) potential well with different diameters of nanoparticles of 35 nm (■), 45 nm (●), 50 nm (▲) and 55 nm (▼). The laser fluence applied in this figure is 4×10^8 W/m^2
